# Epidemiology of overweight and obesity in school children and adolescents in Cameroon: a comparative study in urban and semi-urban settings

**DOI:** 10.11604/pamj.2025.51.87.45733

**Published:** 2025-08-05

**Authors:** Chris Nadège Nganou-Gnindjio, Patrick Yvan Tchebegna, Loïc Alban Mbosso Tasong, Jan René Nkeck, Doris Bibi Essama, Joseph Kamgno

**Affiliations:** 1 Faculty of Medicine and Biomedical Sciences, University of Yaoundé 1, Yaoundé, Cameroon

**Keywords:** Overweight, obesity, children, adolescents, Cameroon

## Abstract

**Introduction:**

over the past three decades, the number of overweight children in Africa has doubled. We aimed to determine the prevalence of overweight and obesity in children and investigate the associated factors in urban and rural areas of Cameroon.

**Methods:**

this was a cross-sectional study on adolescents and children aged between 3 and 19 years, recruited at 22 multi-staged randomly selected schools in Yaounde (urban setting) and Mbouda (semi-urban setting), Cameroon, from December 2017 to June 2018. We collected socio-demographic data, relevant history, and anthropometric measurements. Overweight and obesity were diagnosed according to the International Obesity Task Force for children and adolescents. Associated factors were assessed through multivariate analysis with logistic regression and presented with their OR and 95% confidence interval.

**Results:**

a total of 2398 students were included in the study. The median age was 15 years (IQR 12-17 years). The prevalence rates of overweight and obesity were 27.2% and 9.8% in the semi-urban area vs 27.3% and 30.4% in the urban area (p< 0.001). The prevalence of abdominal obesity was 19.7% in the semi-urban area vs. 15.2% in the urban area (p=0.004). The factors associated with overweight and obesity were increased waist circumference (aOR = 1.03, 95% CI = 1.02 - 1.05, p <0.001), an urban residence (aOR = 2.57, 95% CI = 1.84 - 3.59, p <0.001), increased number of meals per day (aOR = 1.11, 95% CI = 1.01 - 1.23, p =0.034), motorised transport to school (aOR = 1.49, 95% CI = 1.20 - 1.85, p <0.001).

**Conclusion:**

the prevalence of childhood obesity is greater in urban than semi-urban areas of Cameroon. There are many factors associated with the onset of this pathology in our setting, the control of which could be the subject of prevention.

## Introduction

The incidence of overweight and obesity is rising rapidly, particularly among children, making it a major public health problem worldwide [1,2]. It has been estimated that more than 22 million children under the age of 5 worldwide are severely obese, and one child in ten is overweight [3]. Results from a meta-analysis based on 450 nationally representative surveys from 144 countries showed that the overall prevalence of overweight and obesity increased from 4.2% in 1990 to 6.7% in 2010 among preschool children [4].

In sub-Saharan Africa, malnutrition remains frequent, and Cameroon is no exception to this reality [3]. Tchoubi *et al*. found a prevalence of overweight and obesity in Cameroon in 2015 of 8% [4]. Another study conducted in Cameroon by Ebouki *et al*. found a prevalence of overweight in children of 9.1% and in adolescents of 12.4%, while the prevalence of central obesity in children and adolescents was 22.2% and 9.2% respectively [3]. According to the Multiple Indicator Demographic and Health Survey (EDS-MICS) performed in Cameroon in 2011, the national prevalence of overweight and obesity in children was 6% [5,6].

Obesity is associated with cardiovascular, metabolic, and psychological complications and risk of premature mortality in adulthood [5]. The Body mass index (BMI) curves allow for identifying children at risk and when a child is identified as having a real risk of obesity, simple preventive measures, adapted for each subject, could avoid a development toward massive obesity [1].

The rapid increase in the prevalence of overweight and obesity in children/adolescents and its potential impact on morbidity and mortality in childhood and adulthood underscores the importance of identifying critical periods for prevention, populations at risk, and understanding the factors driving excessive weight gain in our context. With this perspective, we aimed to determine the prevalence of overweight and obesity in children and investigate the associated factors in urban and rural areas of Cameroon.

## Methods

**Study design and setting:** this study was conducted in urban (Yaoundé, Centre Region) and semi-urban (Mbouda, West Region) Cameroon. Yaoundé is the second-most populous city in the country. It covers 304 km^2^ with an estimated population of 2.8 million inhabitants in 2014, an average density of 9210 inhabitants per km^2^. The Mbouda municipality, the capital of the Bamboutos division, extends over the central and south-eastern parts of the division. Mbouda has 437 km^2^ over a mountainous landscape whose highest point is located in the Bamboutos Mountains. The total population of Mbouda council is currently around 140,000 inhabitants, with 320 inhabitants per km^2^. We conducted a cross-sectional study among 2398 (1020 semi-urban and 1378 urban) Cameroonian children and adolescents of both sexes, aged 3-19 years, enrolled in 22 randomly selected public and private nursery, primary and secondary schools. The recruitment started on the 08^th^ January 2018 and ended on the 21^st^ May 2018. We conducted a multi-stage cluster random sampling consisting of 1) a census of all primary and secondary nursery schools in each zone and a census of pupils per school. 2) ranking these schools according to school fees; 3) random selection of schools per zone until the target sample size, with an additional 25%, is reached.

**Participants:** all children and adolescents aged 3-19 years were invited to participate through a typed letter of informed consent to their parents or legal guardians to consent for their offspring to be enrolled in the study. The consent was written in both cases.

**Sample size estimation:** the minimum sample size for each study group was estimated using a formula derived from the power calculation formula for comparing two proportions. According to the DHS-MICS, in Cameroon, in 2011, the national prevalence of overweight and obesity among children was 6%. Our study used this prevalence as a mean value to detect a twice as high prevalence in urban school children and adolescents (p1=8%) compared to those in semi-urban schools (p0=4%). With a type I error of 5%, two-tailed tests and a power of 80%, and after correcting for the 10% non-response usually associated with field surveys, we obtained a minimum sample size of 674 pupils for each study group (1348).

**Data measurement:** data were collected using a structured, pre-tested pilot questionnaire. Information letters, consent forms, and questionnaires were given to each class and placed in each kindergarten and pupil's school bag. In contrast, secondary school students were given their letters to give to their parents or legal guardians. Parents who agreed to their child's participation were asked to collect these documents, read the information letter, sign the informed consent forms, complete the questionnaire and return them to their child's backpack. Adolescents were allowed to complete the questionnaire with the help of their parents or legal guardians. These documents were collected by the class teachers and given to our research team. A copy of the signed informed consent was returned to the parents through the same channel. The questionnaire contained personal and social data on the child (date of birth, birth weight, eating habits including early feeding, physical activity during school and leisure time, means of travel to school, sleeping habits, electronic and television use habits, and receipt of pocket money), their mother (age, education level, smoking and alcohol habits, weight and height), and their father (education level). The teacher confirmed physical activity in school.

**Anthropometric variables:** anthropometric variables were measured according to current standards by trained interviewers. Weight was measured to the nearest 100 g using a Seca® scale, with the student's shoes, coat and other heavy outer clothing removed. Height without shoes was measured to the nearest 0.1cm using a portable stadiometer, with the subject standing with their back against the vertical base of the board, heels together. Height and weight were used to calculate BMI as body mass (kg)/square of height (m^2^) [7]. All measurements were taken once using the same brand of scale and stadiometer. The scales were readjusted to zero after each measurement. Waist circumference (WC), measured in centimetres (cm), was measured with a non-stretchable tape at a point midway between the costal margin and the anterior superior iliac spine. Hip circumference, measured in cm at a point passing through both greater trochanters and wrist circumference, in a seated position with the tape measure passing over Lister's tubercle of the distal radius and distal ulna.

**Choice of reference tools and grouping thresholds:** BMI was plotted against the WHO body mass index reference curves for ages 0-5 years and 5-19 years to determine the participant's weight status. Obesity was defined according to WHO recommendations as a body mass index (BMI) > 3 Z-score for children under five years of age and a BMI > 2 Z-score for people aged five years and over [8]. Abdominal adiposity (central obesity) was the waist-to-height ratio (WHtR = waist circumference in cm divided by the waist circumference in cm).

**Statistical analysis:** continuous variables were presented with the median and the interquartile range (IQR), while categorical variables were presented with the proportions. A statistical description of the observations was first performed. Then, chi-square tests were used to determine the associations between the variables of interest (overweight, obesity and central obesity) and the explanatory factors. Implementing an ordered multinomial logit model determined the association between BMI levels and the associated factors. An adjacent, non-parallel multinomial logistic model was applied to calculate the odds ratios (ORs) of one mass body level to another. In addition, a multiple logistic regression model was implemented to assess the association between central obesity and explanatory factors. For variable selection, we used the bottom-up and top-down selection algorithms. All analyses were performed with R v3.4.4 ("The R Foundation for Statistical Computing"), and a p-value at the 5% level was considered the statistical significance level.

**Ethical considerations:** ethical approvals were obtained from the Institutional Ethical Review Board of the Faculty of Medicine and Biomedical Sciences of the University of Yaounde 1, Cameroon. Children and adolescents were included only if their parents or legal guardians signed the information letter and informed consent form. The study was conducted per the principles of biomedical research as stated in the Declaration of Helsinki [9].

## Results

**Baseline characteristics of the study population:** a total of 3,000 children and adolescents were approached. The student study included two thousand three hundred ninety-eight (61.0% female). As shown in Table 1, the median age of the study population was 15 years (IQR 12-17 years). The average age of those living in urban areas (13.5 ± 4.4 years) was lower than those living in semi-urban areas (15.0 ± 3.5 years). As shown in Table 2, the most common age group was 15 to 19 years (61.1%).

**Table 1:** descriptive statistics of quantitative variables

	Minimum 1st	quartile	Median 3rd	quartile	Maximum
Age (Years)	3.0	12.0	15.0	17.0	19.0
BMI (kg/m2)	11.1	17.8	20.3	22.7	42.7
Waist Circumference (cm)	36.0	64.0	70.0	76.0	128.0
Hip Circumference (cm)	41.0	73.0	86.0	96.0	180.0
MUAC (cm)	13.0	20.0	24.0	26.0	38.0
Wrist Circumference (cm)	9.0	14.0	16.0	17.0	26.0
Daily Pocket Money (CFA Francs)	0.0	100.0	200.0	300.0	3000.0

BMI: body mass index; MUAC= 1£ =736.57 CFA Francs

**Table 2:** descriptive statistics of the sample

Characteristics	Number (%)	Characteristics	Number (%)
**Place of residence**		**Sweet drink consumption (times per week)**	
Semi-urban	1020 (42.5)	<3	1538 (64.1)
Urban	1378 (57.5)	≥3	860 (35.9)
**Gender**		**Daytime sleep (number of hours per day)**	
Female	1463 (61.0)	< 1 h	928 (38.7)
Male	935 (39.0)	1 – 2 h	841 (35.1)
**Age**		2 – 3 h	286 (11.9)
[3 – 7[	204 (8.5)	> 3 h	343 (14.3)
[7 – 11[	263 (11.0)	**Birthweight**	
[11 – 15[	465 (19.4)	Poor (<2.5 kg)	285 (11.9)
[15 – 19[	1466 (61.1)	Normal (2.5-3.5 kg)	962 (40.1)
**Number of meals per day**		Excess (>3.5 kg)	430 (17.9)
1	46 (1.9)	**Daily screen time**	
2	295 (12.3)	<3 h	1891 (78.9)
3	818 (34.1)	≥3 h	507 (21.1)
4	728 (30.4)	Ketchup additions in meals	239 (10.0)
5	511 (21.3)	Salt additions in meals	750 (31.3)
**Fruit consumption (times per week)**		Aroma additions in meals	804 (33.5)
<3	1067 (44.5)	Mayonnaise additions in meals	1070 (44.6)
≥3	1331 (55.5)	Nibbling practice	1483 (61.8)
**Travel to school**		Sport practice at school	2222 (92.7)
Walk	923 (38.5)	Sports practice outside of school	1380 (57.5)
Motorised (Motorcycle/taxi/ car/school bus)	1475 (61.5)	Employment of a housekeeper	461 (19.2)
		Practice of video games	897 (37.4)

h: hour; Kg: kilogram

**Figure 1:**
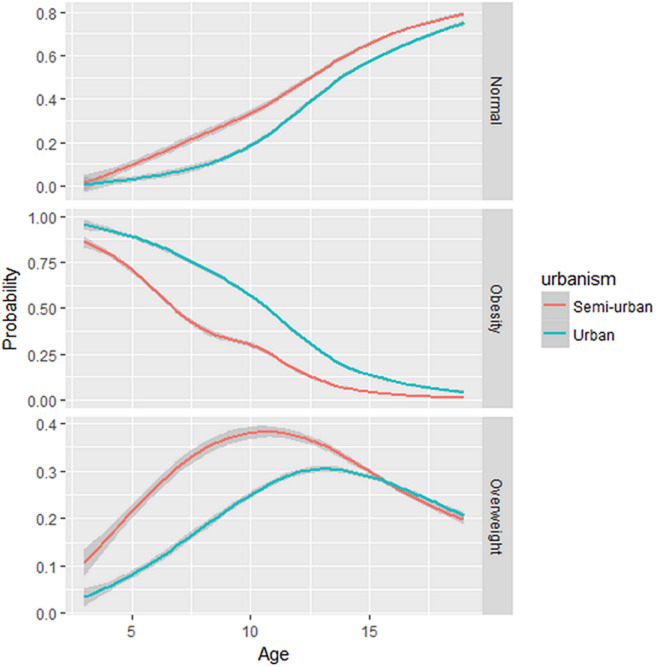
probability of overweight/obesity by age and place of residence; the lines show the mean estimates of the probability of being overweight/obese according to age and place of residence; the red line represents the probability curve in semi-urban areas, and the blue curve in urban areas

**Prevalence and determinants of overweight and obesity:** Table 3 shows that there were more obese people in urban than semi-urban areas (30.4 vs. 9.8%; p < 0.001). After univariate analysis, the factors significantly associated with overweight/obesity in the study were residence in an urban area, age, birth weight, means of transport to school, number of meals per day, number of hours of napping per day, daily screen time and addition of salt and flavouring to meals. In multivariate analysis, children/adolescents were more likely to move from a normal weight status to an overweight status when the number of daily meals was increased by one unit (aOR = 1.11, 95% CI = 1.01 - 1.23, p = 0.034). The odds of going from normal weight to overweight increased with waist circumference (aOR = 1.03, 95% CI = 1.02 - 1.05, p <0.001), motorised transport to school (aOR = 1.49, 95% CI = 1.20 - 1.85, p <0.001). Furthermore, overweight children/adolescents were 2.57 times more likely to become obese in urban areas than in semi-urban areas (aOR = 2.57, 95% CI = 1.84 - 3.59, p <0.001). Increasing age (aOR = 0.810, 95% CI = 0.77 - 0.85, p <0.001) and wrist circumference (aOR = 0.758, 95% CI = 0.67 - 0.85, p <0.001), respectively, decreased the odds of becoming obese compared to those who were overweight. Figure 1 shows that the probability of being obese decreases with age. The probability curve for being obese in urban areas dominates that for being obese in semi-urban areas.

**Table 3:** distribution of overweight, obesity and central obesity

Characteristics	Weight Status				Central obesity	
	Normal weight n (%)	Overweight n (%)	Obesity n (%)	p-value	Central obesity n (%)	p-value
**Place of residence**						
Semi-urban	643 (63.0)	277 (27.2)	100 (9.8)	<0.001	201 (19.7)	0.004
Urban	633 (45.9)	326 (23.7)	419 (30.4)		209 (15.2)	
**Gender**						
Female	783 (53.5)	371 (25.4)	309 (21.1)	0.738	297 (20.3)	<0.001
Male	493 (52.7)	232 (24.8)	210 (22.5)		113 (12.1)	
**Age**						
[3 – 7[	10 (4.9)	19 (9.3)	175 (85.8)	<0.001	73 (35.8)	<0.001
[7 – 11[	40 (15.2)	61 (23.2)	162 (61.6)		52 (19.8)	
[11 – 15[	189 (40.6)	176 (37.8)	100 (21.5)		60 (12.9)	
[15 – 19[	1037 (70.7)	347 (23.7)	82 (5.6)		225 (15.3)	

## Discussion

Our study aimed to determine the prevalence and factors associated with overweight and obesity in young children and adolescents aged 3-19 years in schools in Cameroon. We showed that the prevalence of overweight was 25.1%, and the prevalence of obesity was 21.6%. Overall, there are more obese children and adolescents in urban areas than in semi-urban areas. Finally, some factors that were significantly associated with overweight and obesity were increased waist circumference, urban residence, increased number of meals per day and motorised transport to school. Increasing age and wrist decreased the odds of becoming obese compared to those who were overweight. The probability of being obese decreases with age. The probability curve for being obese in urban areas dominates that of semi-urban areas. On the other hand, the probability of being overweight is higher in semi-urban than in urban areas among pupils under 15 years of age, and this trend is reversed for pupils over 15.

The prevalence of overweight was 25.1%, and obesity was 21.6%. Studies in other sub-Saharan African populations have reported heterogeneous levels of overweight prevalence: 3.5% in 1016 primary school children aged 6-10 years in urban south-western Nigeria, 11.6% in urban south-eastern Nigeria, 13.4% in the sub-group of 4833 Black children aged 6-13 years in South Africa [10]. Unsurprisingly, the reported figures are much more alarming in developed countries. For example, in the United States, 26.7% of children aged 2-5 years are overweight or obese, 32.7% are aged 2-5 years and 32.6% of those aged 6-11 years in 2009-2010 [11]. The results that we present are closer to the latter. It denotes an exponential growth of obesity among children and adolescents in Cameroon if we go by EDS-MICS results. This can be explained by the fact that the criteria for defining overweight and obesity vary from one learned society to another and are not always homologated in different studies. Secondly, the EDS-MICS included rural areas of the country, which diluted the prevalence. They were minimising certain factors that generate obesity in children, as Nurwanti *et al*. [12] reported.

This study shows that the prevalence of obesity is higher in urban areas than in semi-urban areas (30.4% vs. 9.8%). In comparison, there is no significant difference in the prevalence of overweight (27.2% vs. 27.3%). It has been reported in several regions of the world, and it is now generally recognised that the prevalence of overweight and obesity is usually higher in urban areas. At the same time, underweight is generally higher in rural areas [13,14]. We know that children living in urban areas have unhealthy eating behaviours, such as increased consumption of sugar-sweetened beverages that predispose them to weight gain [15]. All this implies the need for central services to focus on campaigns to revive the consumption of fruit and vegetables in families to avoid a definite rise in obesity and morbimortality in countries already on limited income.

Concerning factors such as an increased number of meals per day, it should be noted that the quantity of food consumed is proportionally linked to the quantity of reserve stored, especially in people with a sedentary lifestyle. Motorised transport to school promotes a sedentary lifestyle and reduced physical activity, resulting in a positive caloric energy balance and weight gain, leading to obesity/overweight. All these factors are generally encountered in urban areas, where household incomes are generally higher than in semi-urban or rural areas.

Given the large number of children and adolescents affected, there is an urgent need to modify the current Cameroonian education system by incorporating mandatory courses on physical activity and healthy diets. These policies can contribute to the establishment of sustainable health and education systems that could help transform the current obesogenic environment of Cameroonian children and adolescents into one that encourages healthy eating habits and physical activity, thereby reducing the alarming prevalence of overweight and obesity. They can be applied both generally and to the specific population of our study.

The first limitation of our study was that not all regions of the country were represented; thus, these results do not apply to all children in Cameroon. Furthermore, as this was a cross-sectional study, it was not possible to control for certain factors that might have biased our estimates or results towards the absence or presence of association. Also, due to the design of this study, it is impossible to infer causality or disentangle bidirectional relationships. Nevertheless, this study provides us with a comparison of weight status in urban and semi-urban Cameroon. In addition, our large sample size ensures a certain statistical power.

## Conclusion

The prevalence of childhood obesity is greater in urban than semi-urban areas of Cameroon. There are many factors associated with the onset of this pathology in our setting, the control of which could be the subject of prevention. It will be interesting to modify the current Cameroonian education system by incorporating mandatory courses on physical activity and healthy diets.

### What is known about this topic

- The incidence of overweight and obesity is rising rapidly, particularly among children, making it a major public health problem worldwide;
- The national Cameroon prevalence of overweight and obesity in children was 6% in 2011;
- Overweight and obesity in children/adolescents are potential risk factors of morbidity and mortality in childhood.

### What this study adds

- The prevalence of childhood obesity is greater in urban than semi-urban areas of Cameroon;
- The odds of going from normal weight to overweight increase with waist circumference, motorised transport to school and number of daily meals;
- Increasing age and wrist circumference decreased the odds of becoming obese as compared to being overweight.
